# Folic acid prescription and suicide attempt prevention: effect of past suicidal behaviour, psychiatric diagnosis and psychotropic medication

**DOI:** 10.1192/bjo.2023.549

**Published:** 2023-08-22

**Authors:** J. John Mann, Kwan Hur, Jill E. Lavigne, Robert D. Gibbons

**Affiliations:** Department of Psychiatry, Columbia University Irving Medical Center, New York, New York, USA; Center for Health Statistics, University of Chicago, Chicago, Illinois, USA; Center of Excellence for Suicide Prevention, US Department of Veterans Affairs, Colombia, South Carolina, USA; and Wegmans School of Pharmacy, St John Fisher College, Rochester, New York, USA; University of Chicago, Chicago, Illinois, USA.

**Keywords:** Suicide, self-harm, cost-effectiveness, folic acid, suicide prevention

## Abstract

We previously showed that folic acid prescriptions for any indication were associated with lower rates of suicidal behaviour. Given that future randomised clinical trials are likely to focus on psychiatric disorders carrying elevated risk for suicide, we now report on the moderating effects of prior suicidal behaviour, psychiatric diagnoses and psychotropic medications on potential antisuicidal effects of folic acid. Data were obtained from the MarketScan Commercial Claims and Encounters databases that cover 164 million insured persons from 2005–2017, from which a cohort of 866 586 patients was derived. Analysis revealed no significant moderation effects on the antisuicidal effect of folic acid. These findings indicate that the potential benefit of folic acid for preventing suicidal behaviour is comparable in psychiatric populations at higher risk of suicide and that it may be additive to any benefit from psychotropic medications.

In previous papers we reported that folic acid prescriptions are associated with lower rates of suicidal behaviour.^1,2^ Suicidal behaviour is usually a complication of psychiatric disorders, of which a major depressive episode is the most common. Folic acid could affect suicide risk because it is linked to both response to antidepressant medications^3^ and the pathogenesis of major depression.^4^ Folic acid could also influence decision-making, social cognition and other aspects of the stress–diathesis model of suicidal behaviour. Our findings suggest that folic acid supplementation could lower rates of suicidal behaviour in the general population, but do not address its potential in populations at higher risk of suicide. Clinical trials of its efficacy are likely to be confined to higher-risk populations because their higher base rate of suicidal behaviour increases statistical power and because their clinical need is greater. To guide such future research, we have now determined whether history of prior suicidal behaviour, a known psychiatric diagnosis or receiving a prescription for psychotropic medication moderate the association between folic acid and suicidal events.

## Method

Complete details of our study are available in a previous paper.^2^ In brief, we used the MarketScan Commercial Claims and Encounters databases,^5^ which included in-patient, out-patient and prescription claims from more than 100 insurers in the USA (164 million unique insured-person observations between 2005 and 2017). Codes from ICD-9-CM and ICD-10-CM were used in service claims to identify suicide attempts and self-harm (including deaths by suicide after a medical claim), as well as diagnoses relevant to suicide risk or folate deficiency. Diagnoses relevant to suicide risk were depression, anxiety, attention-deficit hyperactivity disorder, bipolar disorder, schizophrenia, sleep disorders and pain. Prescriptions for two classes of psychotropic medication – antidepressants (selective serotonin reuptake inhibitors, serotonin–noradrenaline reuptake inhibitors and tricyclics) and antipsychotics – were identified. The data were extracted for the period of 2010 to 2018 for adult patients, 18 years or older, filling a folic acid prescription between 2012 and 2017 (2018 data insured at least 1 year of follow-up).

Patients were followed until disenrollment (including death), suicide attempt or self-harm. The modal folic acid dose (48%) was single-agent 1 mg/day, with dosages ranging from 0.4 mg to 5 mg/day. Data were analysed using a discrete-time survival model,^6^ with patients followed up for 24 months after the index folic acid prescription month, which was designated as month zero and not used in the analysis. Month (categorical) was the unit of analysis. Folic acid was a time-varying treatment variable, so we compared suicidal events during months with and without folic acid prescription coverage within individuals. The study cohort were all treated with folic acid for at least 1 month. Although 24% of the cohort were not treated with folic acid during the follow-up period after the initial month, inclusion of these individuals contributed to estimate of the baseline hazard and therefore minimised the standard error of the treatment effect. Prior suicidal behaviour, psychiatric diagnoses, psychotropic medications (as a group) and folate-reducing drugs (as a group) were included in the stratified analyses as baseline covariates, with the exception of the drugs, diagnoses or condition (i.e. prior attempt) that formed the basis for the stratification. Additionally, psychotropic medications were included as time-varying covariates after the index date. Analyses were stratified on prior suicide attempts, mental health conditions and selective serotonin reuptake inhibitor use. Data analysis was performed using SAS 6.4 for Windows.

## Results

Data on 866 586 patients were collected; 704 514 (81.3%) were female and 90 296 (10.4%) were aged 60 years or older. Hazard rates for folic acid showed a lower rate of suicidal events and did not differ based on prior suicide attempt or based on a diagnosis of a psychiatric disorder or being treated with psychotropic medication (overlapping confidence intervals, see Table 1). Folic acid associations with lower hazard rates for suicidal events were statistically significant in all groups except prior suicide attempters, which had a small sample size (*n* = 1286).

**Table 1:** Comparison of hazard rates in psychiatric subgroups on folic acid

Subgroup	Patients, *n*	Treatment	Months, *n*	Suicidal events, *n*	Rate per 100 000 person-years	Hazard rate (95% CI)
Patients with prior suicide attempts	1283	Folic acid	3563	20	6735.96	0.70 (0.42–1.18)
		No folic acid	13 140	78	7123.32	
Patients without prior suicide attempts	865 303	Folic acid	5 518 034	241	52.44	0.56 (0.47–0.65)
		No folic acid	8 419 200	817	116.40	
Patients with mental health conditions	205 514	Folic acid	1 184 858	183	185.28	0.46 (0.38–0.54)
		No folic acid	2 036 582	687	404.76	
Patients without mental health conditions	661 072	Folic acid	4 336 739	78	21.60	0.53 (0.40–0.71)
		No folic acid	6 395 758	208	39.00	
Patients with SSRI use	131 220	Folic acid	851 320	126	177.60	0.59 (0.47–0.73)
		No folic acid	1 238 118	423	409.92	
Patients without SSRI use	735 366	Folic acid	4 670 277	135	34.68	0.55 (0.45–0.68)
		No folic acid	7 194 222	472	78.72	

SSRI, selective serotonin reuptake inhibitor.

## Discussion

This large-scale pharmacoepidemiological study showed that folic acid prescription is robustly associated with lower suicidal event rates in higher suicide risk populations, variously defined by a prior suicide attempt, or a major psychiatric disorder diagnosis or receiving psychotropic medication.

The importance of these new findings is that they extend the observation of lower rates of suicidal behaviour when receiving prescriptions for folic acid, regardless of indication, to populations at high risk of suicide, where the clinical need for such a treatment benefit is greatest. The mechanism of this effect of folic acid warrants further investigation and is potentially related to brain effects on mood regulation and decision-making. It is notable that this putative benefit of folic acid works independently of current psychotropic medications and thus may enhance suicide prevention benefit for high-risk patients beyond that offered by psychotropic medications. The robustness of our findings supports the merit of designing research clinical trials to test the efficacy of folic acid in reducing suicide risk in high-risk psychiatric populations.

## Data Availability

All data used in this study were obtained from Truven Health as a part of their MarketScan database under license to the University of Chicago.
